# Determination of the dehydration pathway in a flexible metal–organic framework by dynamic *in situ* x-ray diffraction

**DOI:** 10.1063/4.0000015

**Published:** 2020-06-23

**Authors:** Ian M. Walton, Jordan M. Cox, Shea D. Myers, Cassidy A. Benson, Travis B. Mitchell, Gage S. Bateman, Eric D. Sylvester, Yu-Sheng Chen, Jason B. Benedict

**Affiliations:** 1 Department of Chemistry, University at Buffalo, The State University of New York, Buffalo, New York 14260-3000, USA; 2 Center for Advance Radiation Sources, The University of Chicago, Argonne, Illinois 60439, USA

## Abstract

Understanding guest exchange processes in metal–organic frameworks is an important step toward the rational design of functional materials with tailor-made properties. The dehydration of the flexible metal-organic framework [Co(AIP)(bpy)0.5(H_2_O)]•2H_2_O was studied by novel *in situ* dynamic x-ray diffraction techniques. The complex mechanism of dehydration, along with the as-yet unreported metastable structures, was determined. The structural information obtained by the application of these techniques helps to elucidate the important guest–host interactions involved in shaping the structural landscape of the framework lattice and to highlight the importance of utilizing this technique in the characterization of functional framework materials.

## INTRODUCTION

Permanently porous crystalline materials, such as metal–organic frameworks (MOFs), are of particular interest for their multitude of potential applications, which include the sequestration of carbon dioxide and other pollutants,^1–5^ low-pressure fuel storage,^6–8^ chemical separations,^9–12^ catalysis,^13–16^ and molecular switches.^17–21^ Applications for these types of materials generally stem from their ability to uptake, exchange, and release guest molecules through their open pores and the void space those pores create. Many of these applications rely on guest-host interactions between the guest and framework to drive selectivity or catalysis of specific guests.

An interesting subset of MOF materials are MOFs that exhibit structural flexibility in response to various stimuli.^22^ The structural response is often dependent on the MOF and can vary between temperature,^23,24^ guest loading,^25,26^ pressure,^27,28^ and interactions with light.^17,29^ The flexibility of some frameworks has been linked to guest selectivity. In most flexible frameworks, the structural changes offer significant benefits to the bulk properties of the MOF and therefore a more advantageous material.^28^

While structures of frameworks upon uptake or release of these guest molecules have been examined by x-ray diffraction in a variety of MOF materials,^28,30–32^ the processes by which these MOFs undergo their transformations is significantly more elusive. The mechanisms of these transformations and the roles the guest molecules play in the overall structure of the framework are two significant questions that are extremely difficult to answer using typical *ex situ* characterization methods. Instead, *in situ* methods can be used to probe the structural responses of these crystalline compounds to changes in their local chemical environment.

*In situ* x-ray diffraction methods have been employed for analyzing powder samples for many years.^33,34^ However, powder x-ray diffraction lacks the atomic resolution of single-crystal diffraction, which can make it difficult to glean important structural information from powder diffraction data. In an effort to observe the structural responses of single-crystals to external chemical stimuli, several Environmental Control Cells (ECCs) have been reported, which allow the crystallographer to collect x-ray diffraction data on single-crystals under non-ambient chemical conditions.^35–37^ However, several of these devices limit the control over the local chemical environment to static conditions. While this does allow the crystallographer to examine single crystals in a variety of chemical environments, these static environments limit the crystallographer to determining structures that are also static.

This work utilizes and expands upon *in situ* SCXRD techniques developed by the Benedict group to study the flexibiltiy of the [Co(AIP)(bpy)_0.5_(H_2_O)]•2H_2_O (**1**) MOF. Careful analysis of the structural response of (**1**) to the local chemical environment around the crystal has led to significant insights into the role of the structure and guest–host interactions on the flexibility of the MOF. Furthermore, several previously unknown structural congeners were determined, leading to a more detailed view of the complex dehyration process of (**1**).

[Co(AIP)(bpy)_0.5_(H_2_O)]•2H_2_O (**1**) is a metal-organic framework, first synthesized by Zeng *et al.* in 2009, which is composed of cobalt(II) metal centers bridged by 5-aminoisophthalate and 4,4′-bipyridine ligands. This framework was shown to undergo several guest exchange processes while maintaining single crystallinity.^30,38^ As a means to develop a novel *in situ* dynamic experimental methodology for use in characterizing guest exchange processes in MOFs, this material was chosen as a candidate for initial studies. In pursuit of the mechanisms of guest exchange in this material, the dehydration of as-synthesized **1** was characterized by dynamic in suit x-ray diffraction (DIX) techniques.^30^ Herein, we report the determination of the dehydration pathway in **1** and the single-crystal structures of this material as the dehydration process occurs.

## EXPERIMENTAL/METHODOLOGY

### Synthesis of 1

To a 23 ml thick walled screw cap vial, 5-aminoisophthalic acid (AIP, 0.045 g, 0.25 mmol), 4,4′-bipyridine (bpy, 0.039 g, 0.25 mmol), NaOH (0.02 g, 0.5 mmol), and water (15 ml) were added, and the reaction mixture was sonicated for 5 min. Following sonication, CoSO_4_•7H_2_O (0.422 g, 1.5 mmol) was added to the vial, and the reaction mixture was heated to 130 °C for three days. The vial was allowed to cool to room temperature, and violet crystals measuring up to 2.5 mm in length were obtained. Single crystals were mechanically separated from powder impurities.

### Data collection methods

DIX data were collected using two different methods. The first method, called “Redundant Scanning” (rs-DIX), involves the collection of a small set of diffraction images, which yield enough information for a reliable unit cell determination. This process is repeated using the same angular coverage throughout the reaction. The rs-DIX method monitors the changes in the position for a subset of the reflections from the crystal, allowing the crystallographer to visually observe changes in the diffraction pattern as the reaction takes place. One major drawback to this method is the lack of reciprocal space coverage, which eliminates the possibility of determining full crystal structures from rs-DIX data.

The “Unique Scanning” method (us-DIX) utilizes a data collection strategy, which covers unique reciprocal space through the course of the reaction. Typically, these scans consist of a pair of 180° ω scans at two different ϕ angles. The collected frames can then be divided up *ex post facto* into small groups of frames, which yield reliable unit cell data, 10 frames typically being the fewest. These datasets often make possible the determination of full crystal structures. Because of the complementary benefits and drawbacks to both methods, studies of this type benefit greatly from a combination of the two. Therefore, for the purposes of this study, accurate time-resolved unit cell determination was carried out on rs-DIX data, while all determined crystal structures were obtained from us-DIX data.

### Dynamic *in situ* x-ray diffraction

rs-DIX data were collected on a Bruker SMART APEX2 CCD diffractometer installed at the Advanced Photon Source, beamline ID-15-B (radiation wavelength = 0.41328 Å)] and equipped with a gas delivery system, which utilizes a mass flow controller to regulate the flow rate of gases over the sample. The data were collected by the rotation method with a 0.5° frame-width and a 0.6 s exposure per frame.

us-DIX diffraction data were collected on a Bruker SMART APEX2 CCD diffractometer installed at a rotating anode source (Mo Kα radiation, λ = 0.71073 Å) and equipped with the same gas delivery system as described previously. The data were collected by the rotation method with a 0.5° frame-width and a 3.0 s exposure per frame. Each crystal structure was determined from two sets of data (360 frames in each set), nominally covering complete reciprocal space.

Crystals of **1** suitable for x-ray diffraction were selected and mounted directly from the mother liquor onto a glass fiber inside an Environmental Control Cell (ECC) using a minimal amount of epoxy.^37^ Following initial crystal structure determination, the crystals were subjected to a stream of dry nitrogen gas at predetermined flow rates. During the dehydration process, diffraction data were collected in one of the two methods described previously.

### Unit cell determination

Unit cells from this study were determined both manually in 10 frame sets and in an automated manner. Automated unit cell determination was achieved through the use of Automatron, an automation program developed at the University at Buffalo.^30^ This program plugs into the Apex3 suite of programs and indexes diffraction patterns using the standard indexing algorithms provided with the Apex3 software.

## RESULTS AND DISCUSSION

**FIG. 1.**
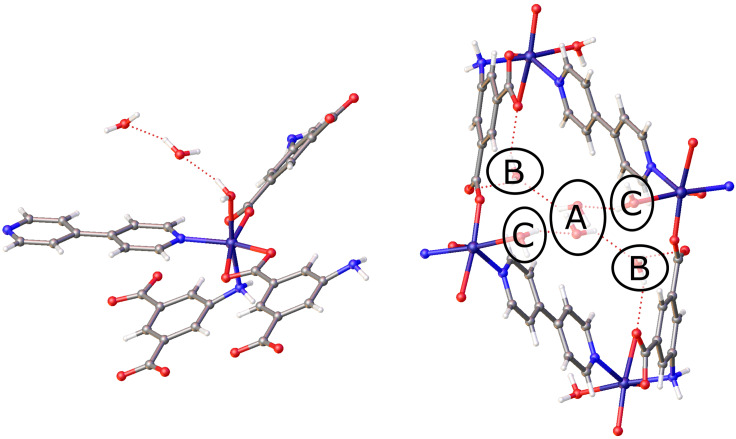
Left: expanded asymmetric unit of **1** showing all ligands attached to one metal center and the two guest water molecules. Right: view of **1** parallel to (010), down the channels created by the pores. Three crystallographically independent guest sites (**A**, **B**, and **C**) are shown. Atoms represented are carbon (gray), nitrogen (blue), hydrogen (white), oxygen (red), and cobalt (dark blue).

The structure of [Co(AIP)(bpy)_0.5_(H_2_O)]•2H_2_O (**1)** contains three crystallographically independent water molecules in the asymmetric unit, one of which is coordinated to the Co(II) metal center. The other two water molecules are located in the interstitial space of the framework. Figure 1 shows the three water binding sites in **1**, designated sites **A**, **B**, and **C**. Previous work by Zheng *et al.* demonstrated, through the use of *ex situ* single crystal x-ray diffraction methods, that the fully dehydrated phase Co(AIP)(bpy)_0.5_ (**4**) could be obtained by heating crystals of **1** under a vacuum at 120 °C for 2 h.^38^ A comparison of the unit cells for these two structures revealed that the crystallographic parameters *b*, *c*, and *β* of the monoclinic cells exhibited modest changes of 0.04 Å, 0.002 Å, and 0.17°, respectively. The observed reduction in the cell volume of approximately 154 Å^3^ upon dehydration was almost entirely a result of the contraction of the *a*-axis by nearly 1.4 Å. Given the large structural change that occurs following the complete loss of the guest water molecules, various DIX experiments were conducted to establish the manner in which the guests were removed from the material and the impact of this process on the host lattice.

**FIG. 2.**
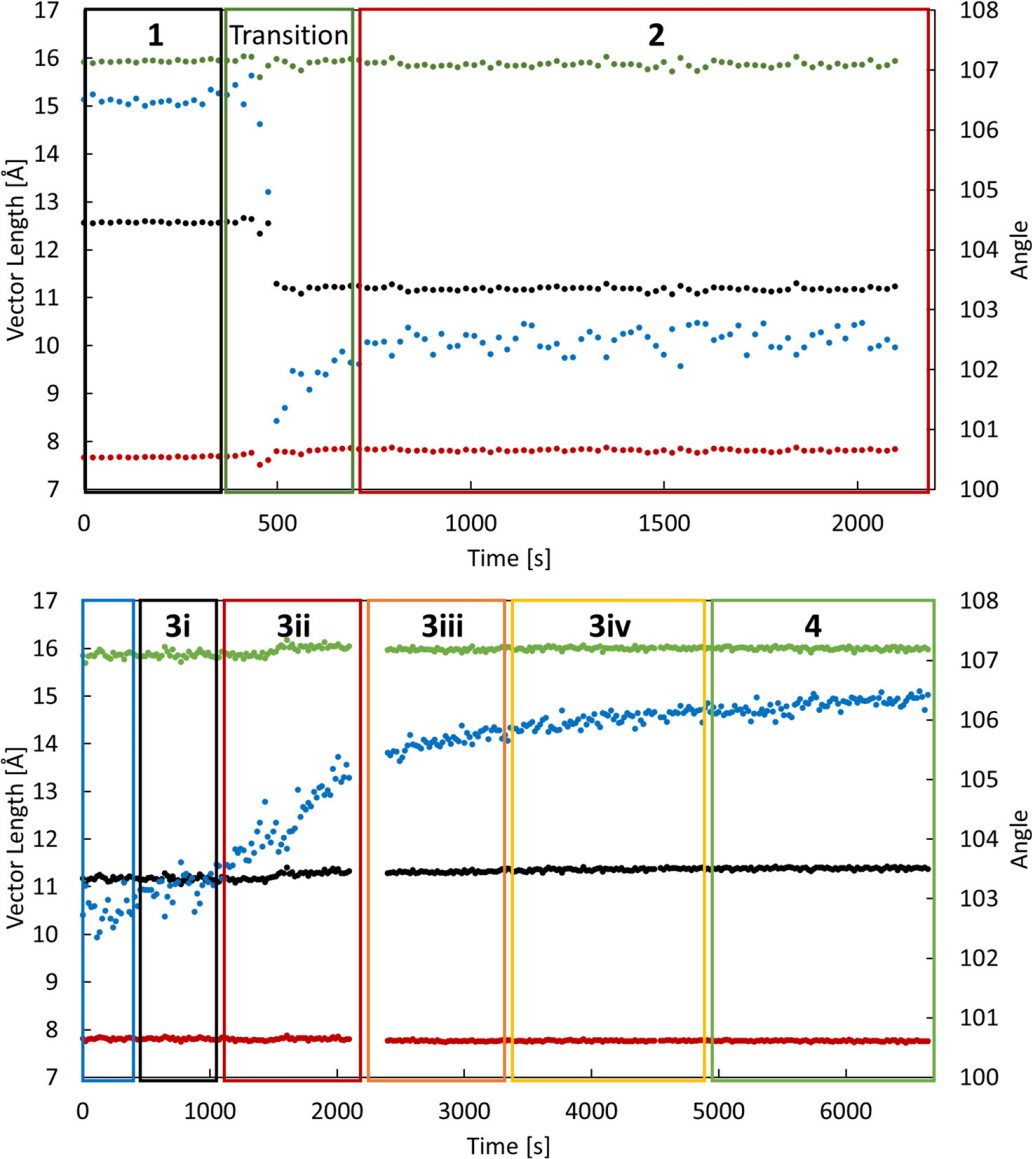
rs-DIX data with an overlay of the regions where the final congeners reside: a-axis (black), b-axis (red), c-axis (green), and β (blue).

Initial experiments found that the complete dehydration of **1**–**4** could be readily accomplished through the application of a flow of dry nitrogen over the sample at room temperature. The mild dehydration conditions left the crystal intact, indicating that single-crystal to single-crystal transition had occurred. The mild dehydration condition was utilized for all other experiments to allow for a controlled dehydration of the sample.

rs-DIX experiments were undertaken throughout the dehydration process to monitor the structural changes in the lattice through unit cell parameters (Fig. 2). The flow rate of dry nitrogen was changed throughout the experiment. After approximately 8 min at a flow rate of 2.5 ml/min, a significant change in unit cell parameters was observed from **1**. While the *b-* and *c-*axes remained relatively constant throughout the change, the *a-*axis decreased by 1.5 Å and β decreased by approximately 5°. The metastable unit cell parameters found at a flow rate of 2.5 ml/min did not match the previously reported anhydrous structure (**2**). To continue the dehydration process, the flow rate of dry nitrogen was then increased to 5 and then 15 ml/min over 100 min. At higher flow rates, the angle of the unit cell gradually increased to 106.5°, resulting in a unit cell approximately equal to **4**. Although the new unit cells derived from the rs-DIX data confirmed the presence of intermediate crystal structures, the short time scales and redundant nature of the scans resulted in datasets with low crystallographic completeness that prevented structure solution and refinement.

To determine the structure of the metastable intermediates along the dehydration pathway, us-DIX experiments were performed on a fresh crystal. In these experiments, the flow rate of dry nitrogen increased at a slower rate (2.5 ml/min steps), with significant stretches of time at each flow rate to allow for complete datasets to be obtained following structure stabilization.

**FIG. 3.**
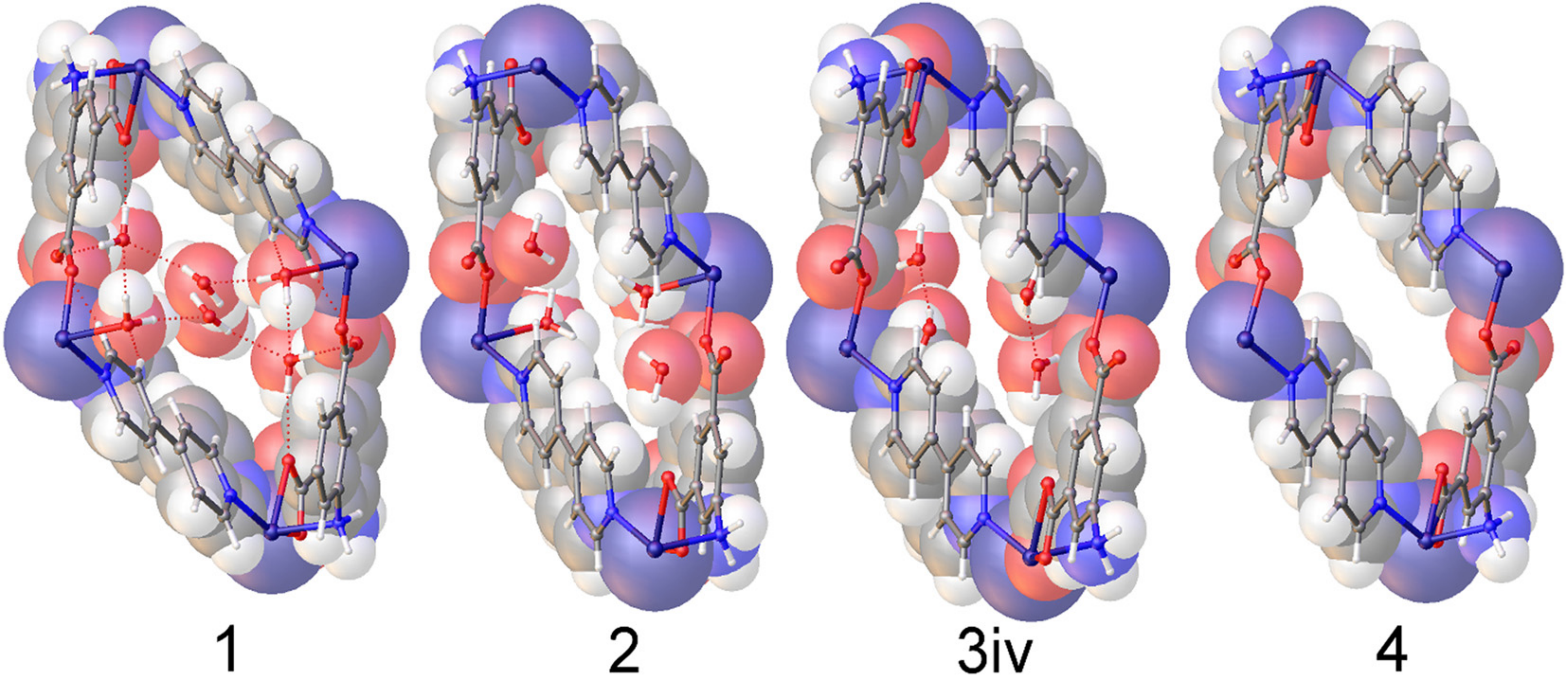
Space-filling models of crystal structures **1**, **2**, **3iv**, and **4**. All models are viewed parallel to (010), the direction of the pores in this framework.

These experiments revealed that the initial rapid transformation of **1**, which involved the simultaneous compression of the *a*-axis and reduction in *β*, results from the loss of a single water molecule to give the dihydrate phase, **2**. Subsequently, several additional structures, designated **3i**, **3ii**, **3iii**, and **3iv**, were determined as *β* increased until the fully anhydrous phase, **4**, was obtained. Structures **3i-3iv** resemble **2**; however, in these structures, the positions of the two crystallographically unique water molecules are only partially occupied. The unit cell parameters of all crystal structures and the occupancies of the three water binding sites in each are listed in Table I. Figure 3 shows the crystal structures of **1**, **2**, **3iv**, and **4** with a space-filling overlay to better illustrate the change in volume throughout the dehydration process. Complete crystallographic data tables for **1**, **2**, **3i**-**3iv**, and **4** are given in the supplementary material.

**TABLE I.** Unit cell parameters and guest site occupancies for crystal structures **1**, **2**, **3i–3iv**, and **4**.

	**1**	**2**	**3i**	**3ii**	**3iii**	**3iv**	**4**
a (Å)	12.6366(8)	11.206(11)	11.224(15)	11.209(6)	11.253(9)	11.303(7)	11.348(9)
b (Å)	7.6914(4)	7.775(7)	7.763(11)	7.794(4)	7.783(6)	7.793(5)	7.755(6)
c (Å)	15.9375(10)	15.842(14)	15.88(2)	15.876(9)	15.929(12)	15.916(10)	15.935(12)
β (°)	106.587(2)	102.39(2)	103.52(3)	104.730(11)	105.304(16)	105.872(12)	106.335(19)
Site A occupancy	1	0	0	0	0	0	0
Site B occupancy	1	1	0.86(2)	0.75(2)	0.66(3)	0.49(2)	0
Site C occupancy	1	1	0.668(15)	0.548(16)	0.37(3)	0.256(13)	0
Total occupancy	3	2	1.53(4)	1.30(4)	1.03(6)	0.75(3)	0

On the basis of the structures obtained from the us-DIX data, the rapid transformation from **1** to **2** coincides with the loss of the water molecule residing at site **A** with sites **B** and **C** remaining fully occupied. As the reaction continues, the crystal structures determined during the slower second step of the dehydration (transformation of **2** to **4)** show a gradual reduction of the occupancy of both sites **B** and **C**. Notably, the crystallographic occupancy of these two sites decreases concurrently but not equally, as opposed to leaving in a stepwise manner.

That site **A** is first to be depleted is not surprising given the relatively poor hydrogen bonding available to water molecules residing at that position. For molecules residing on site **A**, three possible hydrogen bonding interactions are present: site **B** can serve as a hydrogen bond acceptor, site **C** can donate a hydrogen bond, or the site **A** water can donate or accept another site **A** water. Thus, water at site **A** can only form two hydrogen bonding interactions with non-site **A** water, and these interactions are with other water molecules. The relatively weak interaction with the framework likely contributes to the disorder that is observed for the site **A** water.

**FIG. 4.**
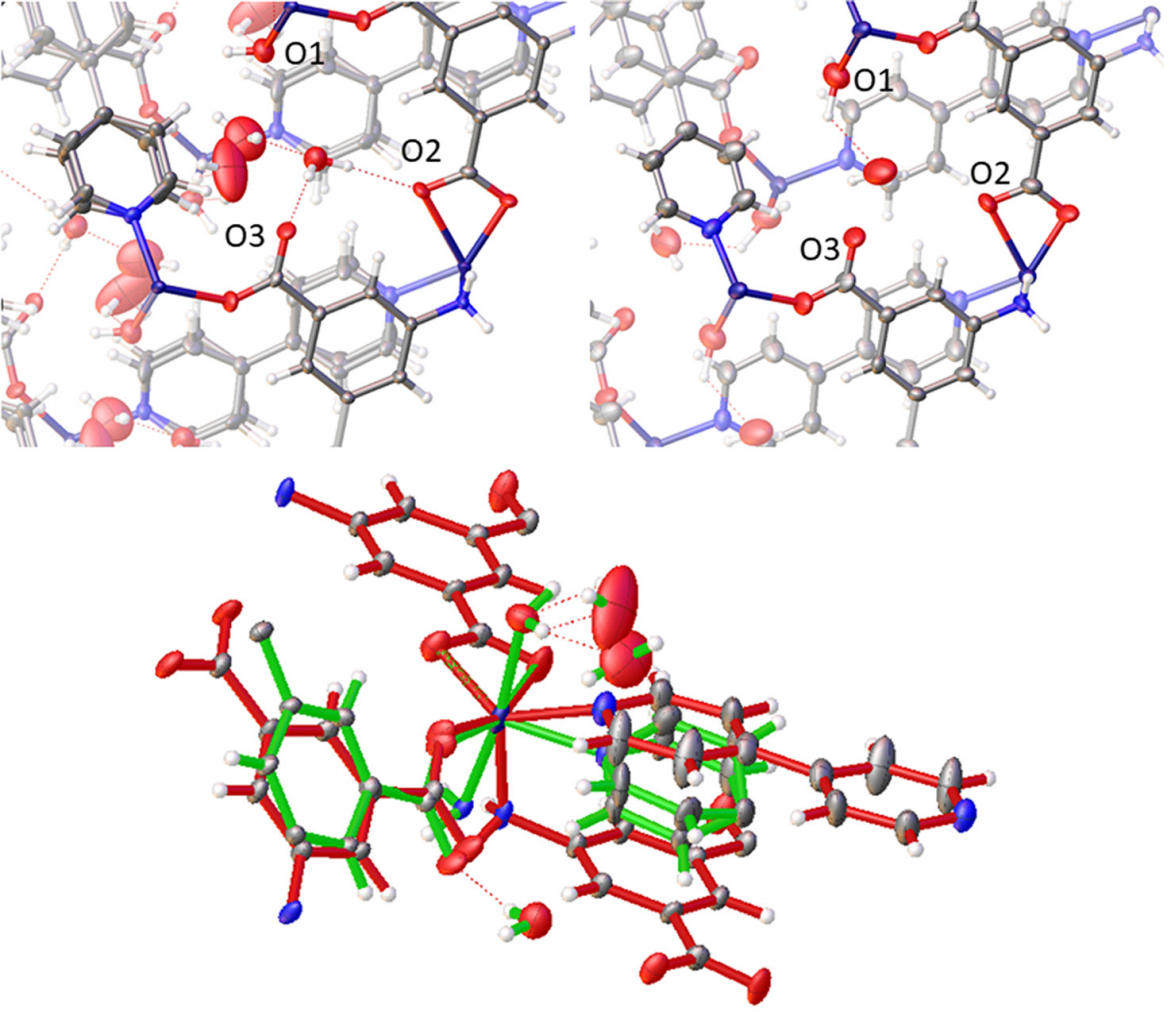
Guest site **B** interactions: **1** (top left) and **2** (top right). O_1_, O_2_, and O_3_ indicate the framework oxygens within the hydrogen bonding range of the guest in site **B**. Bottom: **1** (green) and **4** (red) overlay.

The hydrogen bonding of site **A** stands in stark contrast to that of sites **B** and **C**. For site **B**, the water molecule forms two strong hydrogen bond donor interactions with neighboring carboxylate groups and acts as a hydrogen bond acceptor for water molecules at both site **C** and site **A** (Fig. 4). The water at site **C** is bonded to cobalt and donates a hydrogen bond to water molecules at sites **B** and **C**. The presence of multiple interactions between sites **B** and **C** and the framework contributes to the well-defined positions for water molecules at these sites. That the water molecules at sites **B** and **C** leave concurrently indicates that the relative energies of these two positions are approximately equivalent, although the slightly higher occupancy observed for site **B** relative to site **C** throughout the **2** to **4** transformation may indicate that site **B** is slightly energetically favored relative to site **A**.

**FIG. 5.**
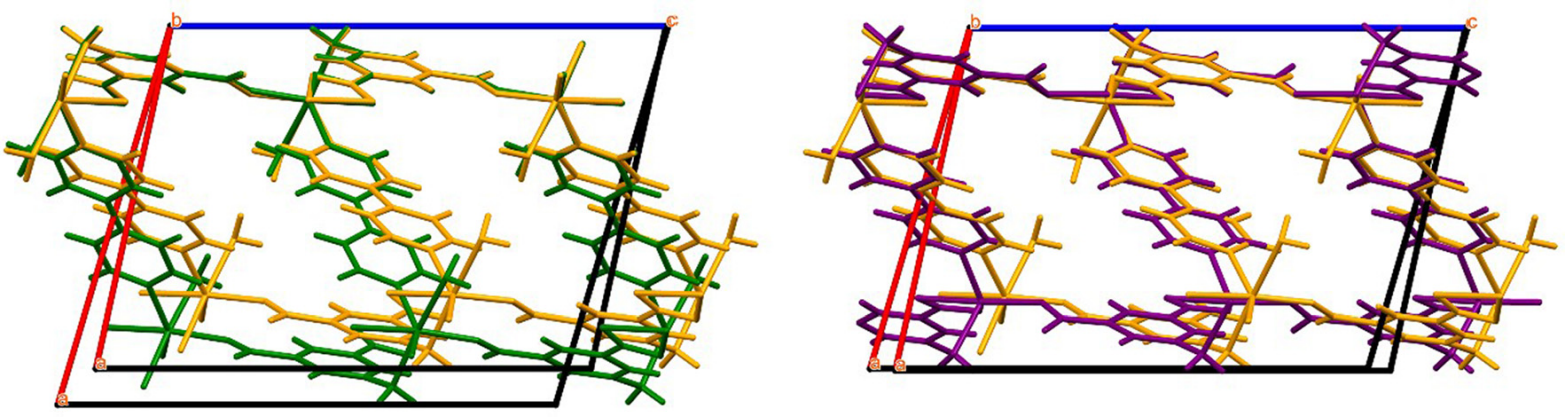
(Left) overlay of structures **1** (green) and **2** (orange). (right) Overlay of structures **2** (orange) and **4** (purple). Only the major disorder species of **1** was used for clarity.

As shown in Fig. 5 the transition between **1** and **2** (loss of **A** site water) results in sheering of the Co-AIP planes, which lie parallel to (100), by approximately 0.80 Å along [001]. When combined with the 1.4 Å contraction of the *a*-axis, the net result is a shift of the Co-AIP plane of 1.65 Å approximately in the [−101] direction. The structural transformation for the transition of **2** to **4** (the loss of **B** and **C** site waters) is more modest and to some extent reverses the change observed in the **1** to **2** transition. This contraction of the pores is responsible for both the reduction of the β angle and the reduction of the *a*-vector. In the **2** to **4** transition, which is characterized by the slow recovery of *β* from approximately 102.5° to over 106°—nearly identical to *β* in **1**, the Co-AIP plane exhibits a sheering of 0.69 Å along $\left[00\bar{1}\right]$. Ultimately, the framework of **4** strongly resembles that of **1.** The most striking difference, the *a*-axis that is 1.5 Å shorter in **4**, is largely absorbed by scissoring of the bipyridine linkers leaving the relative positions of the Co-AIP planes intact (Fig. S1).

The flexible changes of [Co(AIP)(bpy)0.5(H_2_O)]•2H_2_O in response to the dehydration process illustrate a direct relationship between guest occupancy and the structure of the lattice. Analysis of the seven defined congeners throughout the dehydration pathway offers some insights into the direct role of guest–host interactions on the structure of the lattice. In general, the flexibility of the metal center, or the shift of ligands about the metal center, can be attributed to the flexibility of the lattice. However, the role of the guests in sites A, B, and C on the structure of the lattice should not be dismissed. Certainly, the coordination of guest at site C affects the metal center, determining whether the Co(II) center is 5 of 6 coordinate. Further the strong hydrogen bonding potential of the guest at site B may assist in the stabilization of the compressed structure of 2. The change in coordination environment of the Co(II) metal center and the loss of guest at site B likely are the prime contributing factors in the expansion of the channel (Fig. 4).

## CONCLUSIONS

Utilizing dynamic *in situ* SCXRD (DIX) techniques, the dehydration process of [Co(AIP)(bpy)0.5(H_2_O)]•2H_2_O was elucidated in detail, which yielded results impossible to obtain with *ex situ* or static techniques. Several new chemical congeners of the as-synthesized framework were structurally characterized by x-ray diffraction, and the corresponding crystal structures offer many new insights into the role the guest water molecules play in the structure of the framework lattice. The complexity of the observed dehydration mechanism of [Co(AIP)(bpy)0.5(H_2_O)]•2H_2_O, compared to the relatively simple mechanism which would be hypothesized from *ex situ* data serve to highlight the importance of studying dynamic materials using equally dynamic techniques. In addition, studies like this are of great importance as a growing understanding of the interplay between guest and host in these materials can lead to a more concrete understanding of the desirable molecular features for functional MOFs as well as to the rational design of functional frameworks with tailor-made properties.

## SUPPLEMENTARY MATERIAL

See the supplementary material for the table of experimental flow rates, additional images of structures and DIX data, and crystal structure data tables.

## AUTHORS' CONTRIBUTIONS

This manuscript was written through contributions of all authors, and all authors have given approval to the final version of the manuscript. I.M.W. and J.M.C. contributed equally to this work.

## Data Availability

The data that support the findings of this study are available from the corresponding author upon reasonable request. CCDC deposition numbers 1990032–1990038 contain the supplementary crystallographic data for this paper. These data can be obtained free of charge from The Cambridge Crystallographic Data Center via www.ccdc.cam.ac.uk/structures.
